# Outcomes and second-look arthroscopic evaluation after combined arthroscopic treatment of tibial plateau and tibial eminence avulsion fractures: a 5-year minimal follow-up

**DOI:** 10.1186/s12891-015-0769-x

**Published:** 2015-10-21

**Authors:** Tsan-Wen Huang, Chien-Ying Lee, Szu-Yuan Chen, Shih-Jie Lin, Kuo-Yao Hsu, Robert Wen-Wei Hsu, Yi-Sheng Chan, Mel S. Lee

**Affiliations:** Department of Orthopaedic Surgery, Chang Gung Memorial Hospital, Chiayi, Taiwan; Department of Orthopaedic Surgery, Chang Gung Memorial Hospital, Linkou, Taiwan; Chang Gung University, Taoyuan, Taiwan

**Keywords:** Tibial eminence avulsion fracture, Tibial plateau fracture, Arthroscopy, Suture fixation, Arthroscopically assisted treatment

## Abstract

**Background:**

Tibial eminence avulsion fracture often co-occurs with tibial plateau fracture, which leads to difficult concomitant management. The value of simultaneous arthroscopy-assisted treatment continues to be debated despite its theoretical advantages. We describe a simple arthroscopic suture fixation technique and hypothesize that simultaneous treatment is beneficial.

**Methods:**

Patients with a tibial eminence avulsion fracture and a concurrent tibial plateau fracture who underwent simultaneous arthroscopically assisted treatment between 2005 and 2008 were enrolled in this retrospective study. Second-look arthroscopic evaluation and Rasmussen scores of clinical and radiographic parameters were used to assess simultaneous treatment.

**Results:**

Forty-one patients (41 knees) met the inclusion criteria. All 41 fractures were successfully united. All patients had side-to-side differences of less than 3 mm and negative findings in Lachman and pivot-shift tests at their final follow-up. The mean postoperative Rasmussen clinical score was 27.3 (range: 19–30), and the mean radiologic score was 16.5 (range: 12–18). Clinical and radiographic outcomes in 98 % of the patients were good or excellent. There were no complications directly associated with arthroscopy in any patient.

**Conclusions:**

Simultaneous arthroscopic suture fixation of associated tibial eminence avulsion fracture did not interfere with the plates and screws used to stabilize the tibial plateau fracture. It gave the knee joint adequate stability, minimal surgical morbidity, and satisfactory radiographic and clinical outcomes in a minimum follow-up of 5 years and in the arthroscopic second-look assessments.

## Background

Tibial eminence avulsion fracture is less common in adults than in children and adolescents [1]. However, it is one of the most common associated injuries in tibial plateau fractures [2–6]. Formerly, nonsurgical management was recommended for treating isolated Meyers and McKeever type-I tibial eminence avulsion fractures, but the fracture might not be sufficiently secured after the tibial plateau fracture has been stabilized. Thus, it might require additional fixation to provide ligament stability to reduce the probability of developing subsequent osteoarthritis [1]. Nevertheless, combined tibial eminence avulsion and tibial plateau fractures are complex injuries and might lead to difficult simultaneous management.

Because of the advantages of arthroscopically assisted management, which includes directly visualizing intra-articular injuries, a simplified diagnosis, and an accurate reduction of the articular surface, treating meniscal and ligamentous injuries and removing loose fragments yields less surgical morbidity than does traditional arthrotomy [5–8]. Arthroscopic techniques for tibial plateau fractures have recently become the preferred treatment [8–10]. Arthroscopically assisted fixation methods used for the isolated tibial eminence include Kirschner wires, staples, metal screws, and sutures [1, 8–10], as well as a few reported [5, 9] interventions of simultaneous treatment after a short-term follow-up. Outcome improvements and prevention of subsequent osteoarthritis after simultaneous treatment, however, have continued to be uncertain despite the theoretical advantages of arthroscopically assisted fixation methods.

We hypothesized that simultaneous arthroscopic suture fixation using four No. 5 Ethibond Excel (Ethicon US, Johnson & Johnson, Piscataway, NJ) sutures is the ideal technique for providing stable fixation and adequate stability of the knee joint for promoting minimal surgical morbidity, and satisfactory radiographic and clinical outcomes after a minimum follow-up of 5 years. The purpose of the study was (1) to describe a simple arthroscopic suture fixation technique for treating associated tibial eminence avulsion fractures while treating tibial plateau fractures; (2) to report on cruciate ligament, meniscus, and cartilage appearance based on second-look arthroscopic findings; and (3) to analyze the midterm follow-up results of the enrolled patients treated by a single experienced surgeon.

## Methods

This retrospective study was approved by the Ethics Committee and Institutional Review Board (IRB 97-2552B) of Chang Gung Memorial Hospital and all patients provided the signed informed consent.

Patients who had combined tibial plateau and tibial eminence avulsion fractures and had undergone arthroscopically assisted surgery by a single experienced orthopedic surgeon (Y-S.C.) between 2005 and 2008 at our hospital were enrolled. Demographic data and clinical data (number of days of delay before surgery, length of hospital stay, duration between first surgery and second-look arthroscopy, and complications) were reviewed.

All patients underwent standard anteroposterior and plain lateral radiographs. The fracture patterns were evaluated by the emergency room (ER) doctors and the orthopedic trauma consultation duty doctors, and then organized according to the Schatzker classification [11]. Indications for stabilizing the tibial plateau fracture included any varus instability exceeding 10° of a medial tibial plateau fracture at full extension or any lateral plateau fracture with a valgus instability exceeding 10° [12] and an articular step-off exceeding 3 mm or a tibial condylar widening exceeding 5 mm [13]. The associated tibial eminence avulsion fractures were classified as Meyers and McKeever types [14].

Patients who met any of the following criteria were excluded: (a) minimum follow-up of less than 60 months; (b) previous surgery around the affected knee; (c) open, multiple, or pathologic fracture; (d) open growth plates; (e) a severe head injury (initial Glasgow coma scale score <8); (f) severe systemic illness (active cancer, chemotherapy, hemophilia, or a medical contraindication for surgery); and (g) incomplete medical records, radiographic analyses, or clinical functional assessments.

### Preoperative assessment

**Fig. 1:**
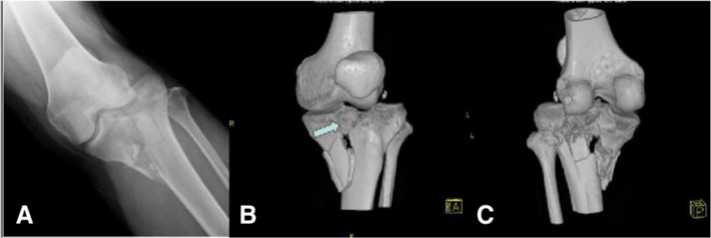
A patient with a Schatzker type V tibial plateau fracture from a motorcycle accident. **a** The plain radiograph shows a bicondylar fracture with a detached tibial eminence (insertion of anterior cruciate ligament [ACL]). **b** A three-dimensional (3D) reconstructed model of computed tomography (CT) scans shows a severely comminuted tibial eminence avulsion fracture (*arrow*) and a depression of the bicondylar articular surface. **c** The 3D model shows huge subchondral and metaphyseal bony defects

Preoperative assessment by the ER doctors included head-injury scoring using the Glasgow coma scale. Sensory and motor functions of the limb were evaluated, and vascular status was determined by checking pulsations of the dorsalis pedis artery and posterior tibialis artery. All the displaced bicondylar tibial plateau fractures were scanned using computer tomography as an additional evaluation and final confirmation of the fracture pattern and associated intra-articular soft-tissue injury (Fig. 1). The condition of local soft tissue around the affected knee was described using the Tscherne classification [15], and damage control techniques were used based on the soft-tissue condition. The readiness of the soft-tissue envelope was determined by the remission of swelling (marked by the return of skin wrinkles), reepithelialization of fracture blisters, and reduction of edema [16, 17].

### Surgical management

**Fig. 2:**
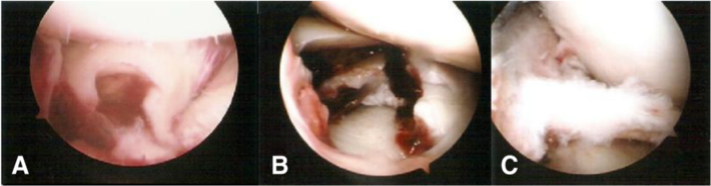
Intraoperative arthroscopic evaluation. **a** A severely comminuted fracture and a depression of the bicondylar articular surface. **b** A lateral meniscus tear. **c** An anterior cruciate ligament (ACL) avulsion fracture. Meyers and McKeever type III. The arthroscopic findings were compatible with the computed tomography (CT) scan findings

The patients were placed supine on the operating table, and complete knee evaluations were done while the patients were anesthetized. The skin around the knee was sterilized and the surgical field was covered with a plastic skin drape. A pneumatic tourniquet was applied to the thigh. The patient was given prophylactic antibiotics between 30 and 60 min before the incision, or 5–10 min before the tourniquet was inflated. It is important to ensure careful fluid extravasation from the knee joint to preclude compartment syndrome. Operative arthroscopy through the anterolateral and anteromedial portals was used to examine the knee after hematomas and loose particles had been evacuated. The capsuloligamentous structures and associated intra-articular lesions were assessed and recorded (Fig. 2).

**Fig. 3:**
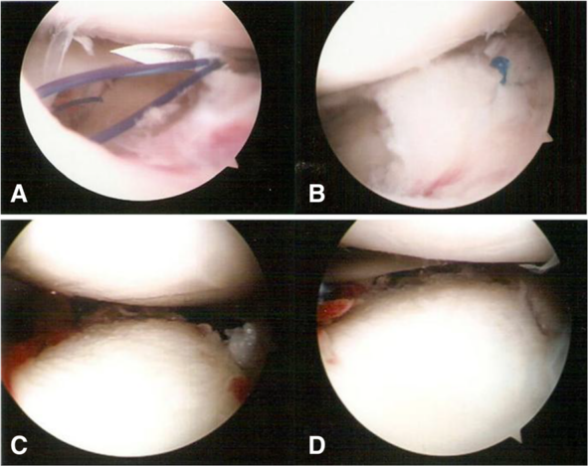
Intraoperative arthroscopic views. **a** and **b** Arthroscopically assisted meniscus suture repair for a lateral meniscus peripheral tear. **c** and **d** The depressed portions of the medial tibial articular cartilage and subchondral bone are elevated using arthroscopically assisted reduction

For associated meniscal injuries, we did a meniscal repair or a meniscectomy before fixating the fracture, because a fracture facilitates access to the meniscus [2–6]. All traumatic peripheral meniscal detachments were repaired using inside-out suture repair if the lesion was located within 5 mm of the meniscosynovial junction [18]. The central radial and longitudinal meniscal tears were treated with a meniscectomy only if they were deemed unstable (Fig. 3-a and b).

For unicondylar fractures (types I-IV), skin incisions were made directly on the medial or lateral side of the fracture, starting from about 1 cm proximal to the articular surface and extending distally for about 8 cm. For a bicondylar fracture (types V and VI), the two incisions were made as far apart as possible. The tibial metaphysis was carefully exposed using minimal periosteal stripping, and care was taken to avoid the arthrotomy. After the subchondral bone and articular surface had been elevated, the resulting bone defect was filled with an allogeneic bone graft or an artificial bone substitute (Fig. 3-c and d). The tibial plateau was accurately reduced and fixated with one (unicondylar fracture) or dual buttress plates (bicondylar fracture). Penetration of the screws to the articular surface was avoided and visually confirmed using an arthroscope.

**Fig. 4:**
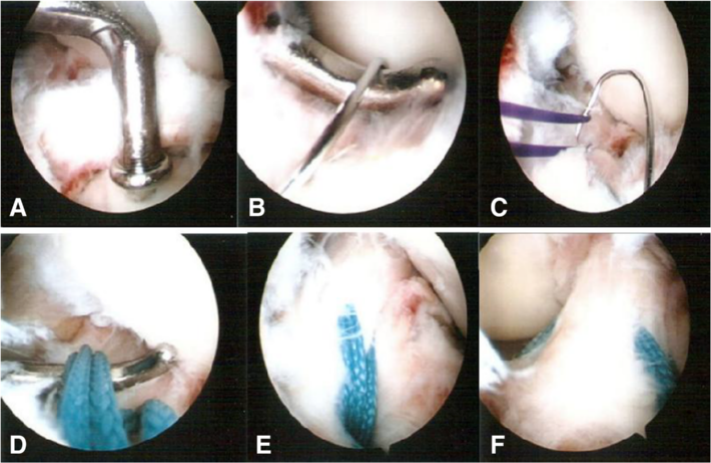
Arthroscopically assisted fixation of a tibial eminence avulsion fracture using the pullout suture technique. **a** The fragment was reduced using an anterior cruciate ligament (ACL) tibial guide. **b** A 26-gauge wire loop was inserted into the knee joint via the medial and lateral tibial bone tunnels, and the diameter of the wire loop was dilated using a probe. **c** The suture hook, loaded with No. 2 polydioxanone sutures (PDSs) as guide sutures, was passed twice—one loop went through the posterior aspect of the ACL, and the second went through the anterior aspect. **d** The actual shuttling of the Ethibond was done using the PDS. The medial ends of each PDS were tied with No. 5 Ethibond loops and retrieved through the medial tibial bone tunnel, passed through the anterior and posterior part of the ACL, and then shuttled into the lateral tibial bone tunnel. Tension was applied to all sutures using a probe to achieve anatomic reduction, restore the ACL to its normal position, and restore the ACL’s normal tension. **e** The four No. 5 Ethibond sutures were individually identified and tied over bone tunnels on the anterior tibial cortex. **f** A good reduction of an ACL avulsion fracture (visualized using arthroscopy)

**Fig. 5:**
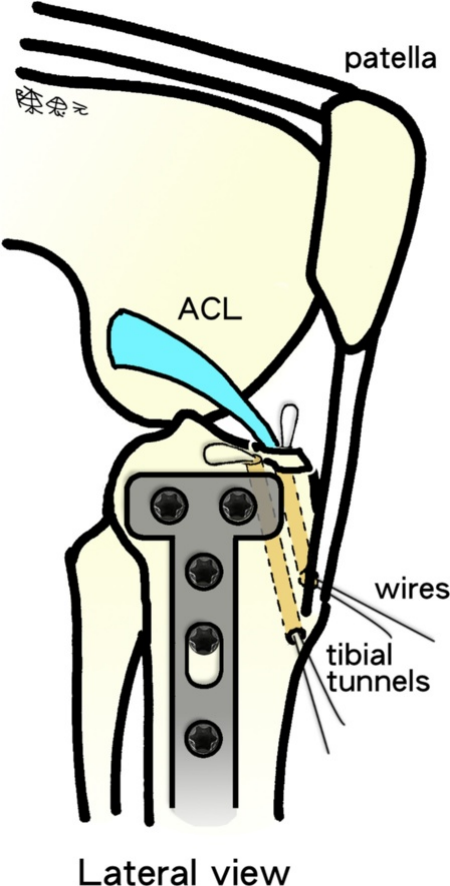
Two 26-gauge wire loops were inserted into the knee joint through the medial and lateral tibial bone tunnels

**Fig. 6:**
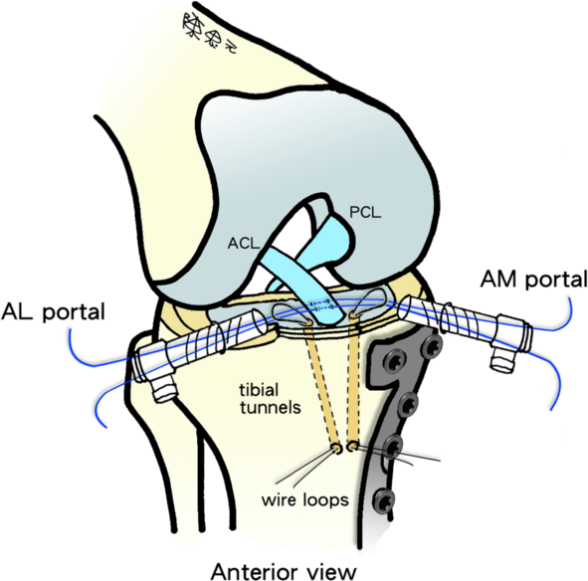
A guide suture being passed through the knee joint through the anteromedial (AM) portal and then through the medial wire loop, the anterior and posterior part of the anterior cruciate ligament (ACL), and the lateral wire loop. (*PCL* posterior cruciate ligament, *AL* anterolateral)

**Fig. 7:**
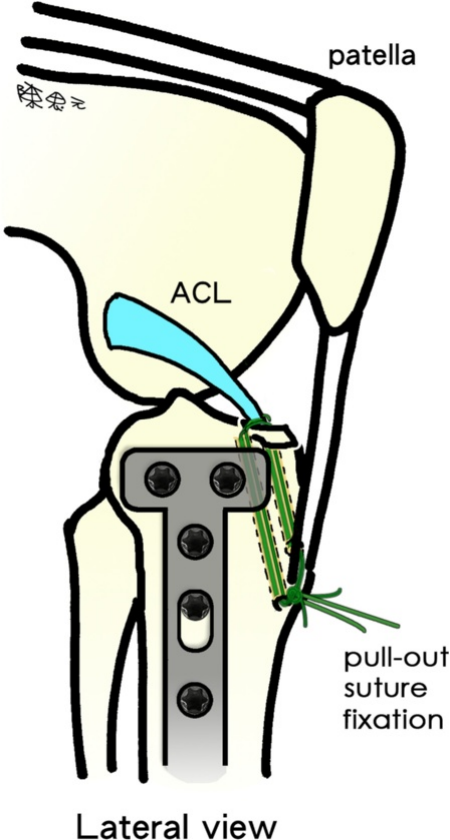
The four No. 5 Ethibond sutures were individually identified and tied over bone tunnels in the anterior tibial cortex. (*ACL* anterior cruciate ligament)

**Fig. 8:**
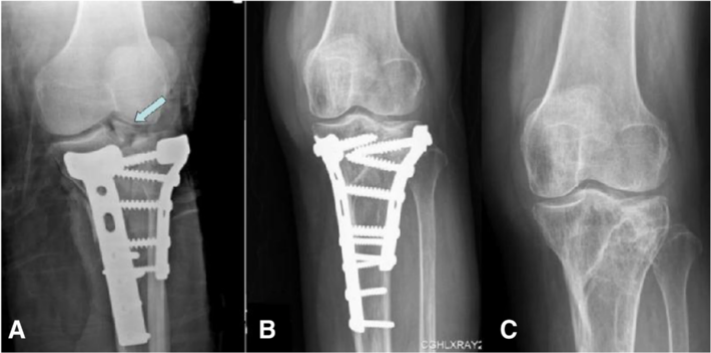
Intraoperative and postoperative plain radiographs (anteroposterior) of the left knee. **a** A residual tibial eminence avulsion fracture on an intraoperative plain radiograph (*arrow*). **b** Postoperative plain radiograph showed a good anatomical reduction of tibial plateau and tibial eminence avulsion fractures after fixation. **c** At the 2-year follow-up, a plain radiograph showed a solid bone union with normal alignment. There were no joint surface depressions or post-traumatic osteoarthritic changes

To treat tibial eminence avulsion fractures, fracture debris and blood clots were debrided for visual access to the avulsed bone fragment and fracture site. An anterior cruciate ligament (ACL) tibial angle guide (Smith & Nephew Endoscopy, Andover, MA) was introduced in the anteromedial portal with the arthroscope placed in the anterolateral portal. The ACL tibial drill guide was used to manipulate and reduce the displaced fracture fragment (Fig. 4-a). Two 2.4-mm Kirschner wires were inserted through the guide from the proximal tibia into the knee joint with a 1-cm bridge of anteromedial metaphyseal cortex. A 26-gauge wire loop was inserted into the knee joint via the medial and lateral tibial bone tunnels (Fig. 5), and then a probe or suture grasp was used to dilate the diameter of the wire loop (Fig. 4-b). The suture hook (Linvatec, Largo, FL), loaded with No. 2 polydioxanone sutures (PDSs) (Ethicon), was used as a guide suture by passing it through the knee joint via the anteromedial portal and then through the medial wire loop, the posterior part of the ACL, and the lateral wire loop. The second guide suture was then passed through the medial wire loop, the anterior part of the ACL, and the lateral wire loop (Fig. 4-c). The medial and lateral wire loops were used to shuttle the No. 2 PDSs through the medial and lateral bone tunnels, respectively (Fig. 6). The actual shuttling of the Ethibond was done using the No. 2 PDSs. The medial ends of each No. 2 PDS suture were tied with No. 5 Ethibond loops and retrieved through the medial tibial bone tunnel, passed through the anterior and posterior parts of the ACL, and then shuttled into the lateral tibial bone tunnel. The knee was extended, and the tibial eminence avulsion fracture was reduced to the fracture bed. Tension was applied to all sutures using a probe to achieve anatomic reduction and to restore the normal position and tension of the ACL (Fig. 4-d). The four No. 5 Ethibond sutures were individually identified and tied over bone tunnels on the anteromedial tibial cortex (Fig. 7). Intraoperative radiographs were routinely taken for all tibial plateau fractures to reconfirm adequate reduction [3, 5, 6] (Fig. 8).

Immediate postoperative care included compressive Cryo/Cuff® therapy (Aircast, Summit, NJ) and a knee brace. Postoperative intravenous antibiotics were given until the suction drainage was removed. Non-weight-bearing range of motion exercises were started with a knee brace once the incisions were sealed and dry. The patients were instructed to remain non-weight-bearing by using crutches or a walker frame until doctors found radiographic evidence of healing. Partial weight-bearing was then allowed for 2 weeks, after which full weight-bearing was permitted.

### Second-look arthroscopic assessment

**Fig. 9:**
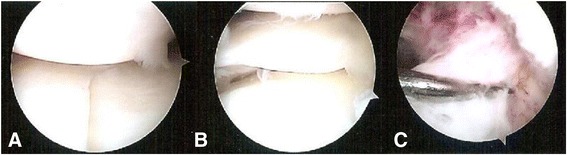
A second-look arthroscopic evaluation. They were compatible with postoperative plain radiographs. **a** A solid union of the fracture without a step-off over the medial and lateral tibial condyles. Good healing of the articular surface with fibrocartilage. **b** A well-healed lateral meniscus after arthroscopically assisted suture repair. **c** A strong and stable anterior cruciate ligament (ACL)

The clinical indications for hardware removal in this study included infection; a broken implant; penetration of a screw into the knee joint; peri-implant fractures; patient complaints and symptoms such as pain, skin irritation, skin changes, allergic reactions to implants, soft tissue compression, etc.; stiffness of the previously fractured limb; and patient requests (e.g., “It doesn’t belong in my body, I simply want to get it out”, “It’s cosmetically disturbing”, etc.) [19]. The risks and benefits of second-look arthroscopic evaluation were explained to all patients. After a patient gave us permission, a second-look arthroscopic evaluation was done in those who chose to undergo this surgery to remove the buttress plates and screws. The strength and stability of the ACL were evaluated using an arthroscopic hook probe, and the cartilage lesion was assessed and then assigned an Outerbridge grade [20] (Fig. 9).

### Clinical and radiological assessment

The Rasmussen system [21] was used to analyze functional (subjective and objective clinical parameters) and radiographic results for treating tibial plateau fractures. This system is widely used in related studies of tibial plateau fractures [3, 5, 6, 22]. A manual Lachman test and a pivot-shift test were used to assess knee stability. To evaluate anteroposterior translation of ligament laxity, side-to-side differences in maximal manual test scores were determined using an arthrometer (KT-1000; MEDmetric, San Diego, CA).

Knee radiographs in standing anteroposterior, standing lateral, and merchant views were taken at 3 months, 6 months, and 1 year postoperatively, and annually thereafter. The radiographic evaluations were examined for articular depression, fracture union, alignment, joint-space narrowing, and degenerative knee changes. The articular depressions were measured using the method described by Di Caprio et al. [9]. Union was defined as the presence of bridging callus on two radiographic views [23]. The severity of degenerative knee changes was graded using the Ahlbäck classification [24]. Each radiograph was reviewed and measured by an independent surgeon using digital radiographs on a computer. The intraobserver reliability was assessed, and the intraclass correlation coefficients (ICCs) were measured according to the method described by Konigsberg et al. [25]. The ICCs of the intraobserver reliabilities of all measurements were 0.726 (range: 0.652–0.939). Because the measurements were judged reliable, measurements made by this blinded observer were used in the analyses.

### Statistical methods

All data were recorded on a Microsoft Excel spreadsheet, rechecked for missing and illogical entries, and then copied into SPSS 13.0 (SPSS, Chicago, IL) for Windows. An independent statistician blinded to the surgical outcomes did all the statistical analyses. A χ^2^ test or Fisher’s Exact test was used to compare categorical data, and a one-way ANOVA test was used for continuous variables. Significance was set at *p* <0.05. A statistical expert was consulted in this study.

## Results

Between 2005 and 2008, there were 82 patients (82 knees) with combined tibial plateau and tibial eminence avulsion fractures who underwent simultaneous arthroscopically assisted treatment. Two patients died of causes unrelated to surgery, eight patients had a minimum follow-up of less than 60 months, four patients had open fractures, four patients had previous surgery around the affected knee, three patients had severe head injuries, three patients had a severe systemic illness, 11 patients refused to undergo second-look arthroscopic surgery, and six patients had incomplete medical records, radiographic analyses, or clinical functional assessments. Therefore, we studied 41 patients (41 knees). The patient population included 21 men and 20 women (mean age: 42.9 years old; range: 21–58 years). The average duration between the first and second operation was 15.3 months (range: 13–27 months). The mean follow-up time was 72.8 months (range: 60–102 months). All patients had been injured in traffic accidents. Twelve fractures were Schatzker type II, eight were Schatzker type IV, six were Schatzker type V, and 15 were Schatzker type VI (Table 1). Eleven of these patients had skin irritation because of the implant, seven patients were disturbed by the cosmetics of the implant. The other 23 patients had no complaints, but they wanted the implants removed.

**Table 1:** Associated lesions and fracture patterns of tibial eminence avulsion fracture

		Meyers and McKeever				Associated lesions		
		Classification				Meniscus	Osteochondral	Collateral
Fracture type	No. of patients	I	II	III	IV	Injury	Loose bodies	Ligament tear
II	12	0	5	6	1	6	2	0
IV	8	1	2	5	0	4	1	0
V	6	0	1	3	2	4	3	1
VI	15	2	2	6	5	11	5	0
Total injuries	**41**	**3**	**10**	**20**	**8**	**25**	**11**	**1**

All data are the number of patients

### Associated lesions in the first surgery

Twenty-six (63.4 %) of the 41 patients had other associated intra-articular lesions: 25 knees had a ruptured or detached meniscus, 11 of which were sutured using the inside-out technique, 14 of which were partially resected, and none of which were totally removed; 11 knees had osteochondral loose bodies, which were removed; and one knee had a grade I medial collateral ligament tear that was treated with a knee brace (Tables 1 and 2).

**Table 2:** Treatment of associated lesions in first and second operations

	1st operation		2nd operation	
Associated lesion	No. of patients	Treatment	No. of patients	Treatment
Meniscus injury				
Peripheral tear	11	Meniscus repair	0	–
Radial tear	9	Meniscectomy	0	–
Flap tear	5	Meniscectomy	0	–
Degenerative tear	0	–	10	Meniscectomy
Osteochondral loose bodies	11	Removed	1	Removed
ACL injury	0	–	2	Shaved
Collateral ligament tears	1	Bracing	0	–

*ACL* anterior cruciate ligament

### Second-look arthroscopic evaluation

Ten of the 41 patients with a degenerative meniscus tear were given an arthroscopically assisted partial meniscectomy; one patient was given an arthroscopically assisted inside-out suture repair for a peripheral tear of the lateral meniscus during the initial surgery, and six of the remaining nine patients were given an arthroscopically assisted partial meniscectomy in the first operation. Osteochondral loose bodies were removed in one knee (Table 2). There was degenerative change of the ACL in two knees; they were arthroscopically shaved (Tables 2 and 3). The articular surface was healing with fibrocartilage over the previous fracture site in 39 patients.

**Table 3:** Second-look arthroscopic evaluation and associated lesions

		Cartilage lesion				Meniscus	ACL	Osteochondral
Fracture type	No. of patients	I	II	III	IV	Injury	Injury	Loose bodies
II	12	2	0	0	0	2	0	0
IV	8	3	1	0	0	2	0	0
V	6	2	1	3	0	1	1	1
VI	15	3	1	1	1	5	1	0
Total injuries	**41**	**10**	**3**	**4**	**1**	**10**	**2**	**1**

All data are the number of patients

*ACL* anterior cruciate ligament

Two patients (4.9 %) had grade III chondral defects. One was a 50-year-old woman with a Schatzker type V fracture. She was treated with osteosynthesis using dual buttress plates. She had a 2-mm step-off with a grade III chondral defect over the medial tibial plateau and a grade III chondral defect on the femoral condyle facing the fracture. The second patient was a 56-year-old man with a Schatzker type V fracture. He, too, had a grade III chondral defect on the medial tibial plateau but only a grade II chondral defect on the femoral condyle facing the fracture.

### Radiological assessment

Radiological results were excellent or good in 98 % of the cases (Table 4). Although the bicondylar fractures (types V and VI) had the worst results, the differences were not significant when compared with unicondylar fractures types II and IV (*P* = 0.209). The fracture types did not have significantly different Rasmussen scores or significantly different satisfactory results. All 41 fractures were eventually united. The mean time to achieve union was 13 weeks (range: 10–17 weeks). The mean preoperative fracture depression was 12.4 mm (range: 8–23 mm). The mean final follow-up fracture depression was 0.3 mm (range: 0–5 mm). There were no cases of severe osteoarthritis with complete bone destruction or a complete loss of space.

**Table 4:** Results of radiographic assessment

Fracture type	No. of patients	Mean clinical score	Excellent	Good	Fair	Poor	Satisfactory result
II	12	16.7 (range: 14–18)	8 (67 %)	4 (33 %)	0	0	100 %
IV	8	17.0 (range: 12–18)	6 (75 %)	2 (25 %)	0	0	100 %
V	6	16.7 (range: 14–18)	4 (67 %)	2 (33 %)	0	0	100 %
VI	15	16.1 (range: 10–18)	4 (27 %)	10 (67 %)	1 (7 %)	0	93 %
Total injuries	**41**	**16.5**	**22 (54 %)**	**18 (44 %)**	**1 (2 %)**	**0**	**98 %**

Unless otherwise indicated, all data are the number of patients (%)

### Clinical assessment

Arthrometer data at 89 N (20 lb) were available for all 41 patients. Postoperatively, all patients had negative findings on Lachman tests and pivot-shift tests. At the final follow-up, all patients had side-to-side differences of less than 3 mm. There were no significant differences in postoperative arthrometer scores compared with the contralateral uninjured limb.

Thirty-one patients had excellent results, nine had good results, and one had a fair result (Table 5). Overall, 40 patients (98 %) had satisfactory results. The differences between results for unicondylar fractures (types II and IV) and bicondylar fractures (types V and VI) were not significant (*P* = 0.873), nor were the differences in Rasmussen scores or satisfactory results. Walking, motion, and stability were good or excellent in all cases. Using the Rasmussen scoring system, 37 patients (90 %) said that they had no pain while walking, three (7 %) reported mild pain while walking, and one (2 %) reported moderate pain while walking. During the final follow-up, the mean active range of motion was 121° (range: 0–130°). One patient had fair results because of postoperative extension lags greater than 10°.

**Table 5:** Results of clinical assessment

Fracture type	No. of patients	Mean clinical score	Excellent	Good	Fair	Poor	Satisfactory result
II	12	27.7 (range: 25–30)	11 (92 %)	1 (8 %)	0	0	100 %
IV	8	27.3 (range: 26–30)	6 (75 %)	2 (25 %)	0	0	100 %
V	6	28.2 (range: 26–30)	5 (83 %)	1 (17 %)	0	0	100 %
VI	15	26.7 (range: 19–29)	9 (60 %)	5 (33 %)	1 (7 %)	0	93 %
Total injuries	**41**	**27.3**	**31 (76 %)**	**9 (22 %)**	**1 (2 %)**	**0**	**98 %**

Unless otherwise indicated, all data are the number of patients (%)

### Complications

Four patients had paresthesia over the lateral calf of the lower leg. Two patients had wound dehiscence over the incision lines. One patient with a Schatzker type VI fracture had wound dehiscence over the medial and lateral surgical incision lines. The wounds healed after antibiotic therapy. Another patient with a Schatzker type VI fracture had wound dehiscence over the medial surgical incision line. Infection and poor soft tissue coverage occurred 21 days after the index procedure. These problems were successfully treated with repeat debridements, rotational gastrocnemius flaps, and split-thickness skin grafts on the medial incision. During the final follow-up, no patient had clinical signs or symptoms of serious complications such as compartment syndrome, knee stiffness, or deep venous thrombosis, nor were any complications directly associated with arthroscopy.

## Discussion

The key finding of this study is that arthroscopically assisted combined treatment of tibial plateau and tibial eminence avulsion fractures provided satisfactory radiographic and clinical results after a minimum follow-up of 5 years. There were no cases of severe subsequent osteoarthritis with complete bone destruction or a complete loss of space. This technique is easy to perform, and it is effective for ensuring satisfactory stability of the knee, useful for fixating tibial eminence avulsion fractures without interfering with the stabilizing plates and screws of the tibial plateau fracture, and effective for providing minimal surgical morbidity.

Our results are comparable to those of Di Caprio et al. [9], who reviewed 21 knees with associated tibial eminence avulsion and unilateral tibial plateau fractures (Schatzker types I, II, and IV). They reported good results using percutaneous screw fixation for the tibial plateau fracture and arthroscopic suture fixation for the tibial eminence avulsion fracture. Our series is larger and we found that this technique was useful and did not interfere with the plates and screws in patients with unilateral (Schatzker types II and IV) and bilateral (Schatzker types V and VI) tibial plateau fractures. We report that arthroscopic suture fixation yielded excellent and good radiographic results and midterm clinical outcomes. Moreover, second-look arthroscopic evaluation detected good stability of the tibial eminence and tension of the ACL, findings comparable to those of studies using an arthrometer.

Most studies have evaluated the success of managing a tibial plateau fracture by looking at radiographic and functional results [26]. The second-look arthroscopic evaluation not only provides a safe, quick, and accurate method of assessing outcomes, but it also provides the only way to show the healing of articular cartilage after a tibial plateau fracture has been treated [27–29]. So far, only three studies have used second-look arthroscopy to directly observe and assess the intra-articular structures after a tibial plateau fracture. Cetik et al. [27] evaluated 12 knees and concluded that the potential for cartilage healing is limited in human beings, even when anatomical reduction succeeds. Cartilage defects were detected over fracture lines and on femoral condyles facing the fracture site. Lee et al. [28] assessed 20 patients with Schatzker type II tibial plateau fractures. They found that the actual condition of the articular cartilage varied significantly, even in patients with a normal joint range of motion and good clinical and radiological results. Ruiz-Ibán et al. [29] studied 13 patients who had undergone combined arthroscopic treatment for a tibial plateau fracture and a meniscal tear. One patient had a grade II lesion with a clear decrease in his activity level and a low adjusted IKDC (International Knee Documentation Committee) score attributable to a chondral lesion. In our study, the second-look arthroscopic evaluation findings for all 41 knees were similar to those in Ruiz-Ibán et al. A 50-year-old woman had a 2-mm step-off with grade III lesions and an unsatisfactory result.

Because of its complexity and associated soft-tissue injuries, tibial plateau fracture is always challenging for orthopedic surgeons [30]. The major treatment objectives for this circumstance are reconstructing articular surfaces, restoring joint congruity, and obtaining knee joint stability after fixation for early motion, a sufficient range of motion for bending and rotation, and the transmission of large muscular loads [3–10, 31–33]. With arthroscopic assistance, the quality of intra-articular reduction and stable fixation without detaching the menisci and preserving the soft-tissue envelope can be achieved and provide satisfactory results and less surgical morbidity [3–8, 27–29]. Concomitant injuries are an important confounding factor: studies using arthroscopic evaluation [2, 7, 8, 27–29, 34, 35] have reported the frequency of associated ligament and meniscal lesions to be as high as 71 %. Although some are merely minor injuries, many of them can compromise the final outcome and can cause major difficulties for simultaneously treating the fracture and the soft-tissue injuries [7–9, 29].

The tibial eminence avulsion fracture is one of the most common concomitant injuries. The effectiveness of various arthroscopic intervention techniques (fixation with Kirschner wires, staples, metal screws, or sutures) has been demonstrated [1, 8–10]. Both metal screw fixation and pullout suture fixation for Meyers and McKeever type III tibial eminence avulsion fractures provide better biomechanical advantages and are more effective for obtaining initial rigid fixation than are biomechanical methods [36, 37]. However, metal screw fixation for isolated Meyers and McKeever type IV fractures is technically impossible; moreover, it is difficult to place the screws when stabilizing tibial plateau fractures using plates and screws [10, 36–38]. In contrast to metal screw fixation, sutures inserted through the ACL base provide secure fixation, even when the fracture fragment is comminuted or small. This method also allows the surgeon to provide the correct amount of tension to the ACL [10, 12]. The simple arthroscopic suture fixation technique for isolated tibial eminence avulsion fractures that we previously reported [10] provides satisfactory radiographic and clinical outcomes. This technique has several advantages: it is simple, it requires no additional surgery to remove the implants, it does not fragment the tibial eminence, and it allows precise repair of bone fragments. In the present study, this technique was also ideal for producing satisfactory radiographic and clinical results when simultaneously treating the associated tibial eminence and tibial plateau fractures.

This study has some limitations. First, the study was not randomized. Second, it was limited by its retrospective design, small sample size, and lack of a control group. The tibial plateau fracture type might interfere with the final results; therefore, it is better to enroll more patients in each group to see the differences between different tibial plateau types. However, it is the largest study focused on the simultaneous treatment of associated tibial eminence fractures when stabilizing tibial plateau fractures. The bias might be reduced because all patients were treated by the same experienced surgeon, with the same surgical technique, and with the same treatment protocol. Third, the clinical follow-up was brief. Several studies [1, 3–9] have reported early- to medium-term results of the arthroscopically assisted osteosynthesis of combined tibial eminence and tibial plateau fractures, but long-term results are worthwhile and warrant investigation to determine whether arthroscopically assisted management yields improved long-term clinical outcomes.

## Conclusion

In conclusion, simultaneous treatment of associated tibial eminence fractures and tibial plateau fractures using arthroscopic suture fixation was technically easy and did not interfere with the plates and screws used to stabilize the tibial plateau fracture. There was no case of severe subsequent osteoarthritis. The radiographic and functional outcomes were satisfactory in a minimum follow-up of 5 years and in the arthroscopic second-look assessments.
